# Pacemaker Related Infective Endocarditis from *Staphylococcus Lugdunensis*: A Case Report

**DOI:** 10.1155/2013/180401

**Published:** 2013-01-22

**Authors:** Michael Ward, Kevin M. Boehm

**Affiliations:** ^1^Department of Emergency Medicine, Henry Ford Wyandotte Hospital, Wyandotte, MI 48192, USA; ^2^College of Osteopathic Medicine, Michigan State University, East Lasting, MI 48824, USA; ^3^College of Osteopathic Medicine, Nova Southeastern University, Lauderdale, FL 33314, USA

## Abstract

*Staphylococcus lugdunensis* is a common skin flora not typically associated with infection. There are, however, several cases reported in the literature of *Staphylococcus lugdunensis* as a causative bacterium of various infections. This paper reports an additional case of pacemaker associated endocarditis with *Staphylococcus lugdunensis* as the causative bacterium.

## 1. Introduction

*Staphylococcus lugdunensis* is a common skin flora that is seldom a causative organism of infection. It is a coagulase-negative staphylococcus that was first described in 1988 and has been associated with various community and nosocomial infections including native/prosthetic valve endocarditis, skin and soft tissue infections, catheter-related infections, ventriculoperitoneal shunt infections, prosthetic joint infections, and pacemaker related infections [1–4]. *S. lugdunensis* is proving to be more virulent than other coagulase-negative staphylococci, which is thought to be related to several of its unique abilities including the production of a toxin-like hemolytic peptide, adhesion promoting molecules, various enzymes, and biofilm formation [2]. It also produces a Von Willebrand bound binding protein that allows it to adhere to the endocardium endothelium lesions [2].

The frequency of *S. lugdunnsis* is likely underreported because it is easily confused with *S. aureus* through *S. lugdunnsis'* ability to produce clumping factors and the fact that many labs do not further isolate out coagulase-negative staphylococci variants [2]. Fortunately, it is often susceptible to most antibiotics already used to treat other staphylococcal infections with little reported resistance. An estimated 25% of strains are capable of producing b-lactamase and even methicillin resistance is rarely reported [2]. 

## 2. Case

A 74-year-old Caucasian female presents to the emergency department of a local community hospital with a chief complaint of diarrhea for the past 3-4 days and a productive cough. All other review of systems was documented as negative. Her medical history was positive for hypertension, TIA, and an unspecified cardiac condition requiring a pacemaker. Her home medications included Bumetanide, digoxin, atorvastatin, carvedilol, lisinopril, and oral potassium supplement. Surgical and social histories were noncontributory. All history was obtained from the patient who was reported as alert and oriented for the duration of the emergency room stay.

On initial presentation, her vital signs were as follows: a blood pressure of 131/84, pulse of 108, respiratory rate of 20, temperature of 38.6, and a pulse oximetry of 94% on room air. She was reported to be in a new onset atrial fibrillation with a rapid ventricular rate. The rest of her examination was positive for the tachycardia with an irregularly irregular rhythm and 2+ pitting edema bilaterally in her lower extremities.

The initial electrocardiogram confirmed an atrial fibrillation with a rate of 112 beats per minute and other nonspecific changes. A posterior-anterior and lateral chest radiograph showed no acute process and stable pacemaker hardware. Initial complete blood count revealed a leukocytosis at 11.9 K/uL (3.8–10.6 K/uL), hemoglobin of 8.2 g/dL (12.0–15.0 g/dL), hematocrit of 25.9% (36–46%), and a platelet count of 42 K/uL (150–450 K/uL). Serum sodium measured 133 mmol/L (135–145 mmol/L), potassium of 5.1 mmol/L (3.5–5.0 mmol/L) with no mention of sample hemolysis, bicarbonate of 18 mmol/L (24–32 mmol/L), and an anion gap of 12. Kidney function testing had a creatinine level of 2.0 mg/dL (0.6–1.2 mg/dL) and a BUN of 41 mg/dL (10–25 mg/dL) with no reported history of kidney failure. She had a lactic acidosis with a lactate of 4.8 mmol/L (0.4–1.8 mmol/L) and the digoxin level was just above therapeutic range at 2.1 ng/ml (0.8–2.0 ng/ml). Troponin I was elevated to 0.11 ng/ml (<0.10 ng/ml) on presentation. Total bilirubin was elevated at 1.8 mg/dL (0–0.3 mg/dL) and the INR was 2.14 (0.80–1.20). Urine sample was nitrite positive with few bacteria reported on microscopic exam, 0–5 WBC/HPF (<10), and 10–20 RBC/HPF (<3). There were also 0–5 squamous cells/HPF and 0–10 granular casts/HPF. 

Overall, this patient presented with a new onset atrial fibrillation with a rapid ventricular rate, acute anemia without signs of active bleeding, non-ST segment elevation myocardial infarction (NSTEMI), acute renal failure, and an elevated bilirubin and INR. Suspected etiology was initially hypovolemia from sepsis confounded by further volume loss via diarrhea. An abdominal ultrasound was performed and was negative for acute pathology. Blood and stool cultures were obtained and a stool for *Clostridium difficile* was negative.

Treatment in the emergency department consisted of fluid resuscitation with 0.9% normal saline (NS) boluses, for a total of 3L of NS in the emergency department as well as 2 units of packed red blood cells were transfused. She received 2 liters of oxygen via nasal cannula and was given 1 gram of intravenous ceftriaxone for UTI. Her four-hour repeat troponin was elevated to 1.97 ng/ml (<0.10 ng/ml) and the lactic acid elevated to 6.0 mmol/L (0.4–1.8 mmol/L). Thyroid studies were ordered and not found to be a contributing factor to the new atrial fibrillation with rapid ventricular rate with a thyroid stimulating hormone (TSH) of 5.72 uIU/ml (0.35–5.50 uIU/ml) and free T4 of 0.88 ng/dL (0.59–1.17 ng/dL). She remained in the emergency department approximately five hours before a bed was available in the step-down intensive care unit. 

The antibiotics were switched to intravenous cefepime 2 grams every eight hours, 250 mg of oral vancomycin every eight hours, and 500 mg of intravenous metronidazole every eight hours on the floor. She was seen by cardiology, nephrology, gastroenterology, hematology, and infectious disease specialists. General surgery was consulted for possible abdominal etiology of sepsis per documentation. CT abdomen without contrast revealed a low density lesion within the spleen, mesenteric edema, questionable bowel wall thickening of the ascending colon, and a right pleural effusion with bilateral patchy consolidations. General surgery opted to continue antibiotics at that time without immediate surgical intervention.

Labs were repeated the following morning. Her lactate had elevated to 11.2 mmol/dL (0.4–1.8 mmol/L) despite resuscitation. The leukocytosis elevated to 17.4 K/uL (3.8–10.6 K/uL), potassium to 5.4 mmol/L (3.5–5.0 mmol/L), bicarbonate of 7 mmol/L (24–32 mmol/L) with anion gap of 23 (8–16). Troponin I elevated to 2.88 ng/ml (<0.10 ng/ml), Cr. to 2.3 mg/dL (0.6–1.2 mg/dL), and INR >19.2 (0.80–1.20). The patient had developed disseminated intravascular coagulation (DIC), likely secondary to sepsis. Transfusion of four units of fresh frozen plasma and six units of platelets were initiated. Hydrocortisone 25 mg intravenously every 6 hours and intravenous Vitamin K 10 mg were administered. The patient ultimately went into acute respiratory failure from the fluids and blood products she received, was intubated by anesthesia, and moved to the intensive care unit. She also became hypotensive and required a continuous infusion of norepinephrine, subsequently developed oliguria, and a right femoral dialysis catheter was placed in case she would later require dialysis. Her blood cultures came back positive for gram positive cocci identified as *Staphylococcus lugdunensis*. Infectious endocarditis was suspected and nafcillin was started. A transthoracic 2D echo was performed and showed large vegetation on the posterior leaflet of the mitral valve. Transesophageal echocardiogram confirmed this finding and further identified additional vegetation on the anterior leaflet of the mitral valve measuring 0.4 cm. The posterior vegetation measured 1.8 cm and was noted to be very mobile. At this point, the CT findings were thought to be secondary to septic emboli.

The laboratory abnormalities began to improve the following day and she was started on rifampin in addition to the nafcillin with all other antibiotics discontinued. Her leukocytosis, lactate, troponin, and creatinine began to trend down, but the decision was made to transfer the patient to a local tertiary center affiliate for the definitive care of pacemaker removal where she was accepted to the medical intensive care unit.

The patient had a 24-day course between the two facilities. Her DIC resolved and she was still in the atrial fibrillation with a controlled ventricular rate during her stay. On hospital day 10, the pacemaker and its leads were removed out of concern, it was the source of infection. It was reported to be implanted on 1/15/2010 and was a Biotronik Cyclos DR with one lead in the right atrium and another in the right ventricle. The leads were sent to lab for tip cultures. The right atrial lead showed rare *S. lugdunensis*, while the right ventricle lead revealed few *S. lugdunensis* that was susceptible to nafcillin. A temporary transvenous pacemaker was immediately replaced; however, this pacemaker was found not necessary and subsequently removed 48 hours later. The patient was still on intravenous naficillin and rifampin and was finally extubated on hospital day 11. Repeat blood cultures were negative for growth after 5 days on hospital days 11, 19, and 20. The discharge summary reported that the nafcillin was discontinued because she began to develop rashes. She was discharged on oral warfarin for her atrial fibrillation. Her kidney function returned to baseline with creatinine of 1.2 mg/dL (0.6–1.2 mg/dL). Subsequent transthoracic echo cardiograms revealed that the mitral valve vegetations were improving. She was discharged to a sub-acute rehab facility with continued intravenous nafcillin therapy and outpatient followup with cardiology and infectious disease.

The patient returned to the original community hospital's emergency department three days after discharge with chief complaint of mental status changes. She was found to have bright red blood per rectum and an anemia of 6.0 g/dL (12.0–15.0 g/dL) with a hemoglobin level of 9.0 g/dL (12.0–15.0 g/dL) reported from the tertiary facility at discharge. The decision was made by family to make the patient not to resuscitate (DNR) status with no desired intubation or mechanical ventilation. The patient was only oriented to person at this time and incapable of making her own medical decisions. She was placed in hospice care and expired two days later. 

## 3. Discussion 

*S. lugdunensis* is as emerging as a pathogen that presents similar to *S. aureus*. It has been associated with several cases of infective endocarditis with an inclination for left sided, native valve related infections [5]. Despite its reported high levels of susceptibility to various antibiotics, medical treatment alone is often unsuccessful and surgical interventions are often required. Jung et al. report an increase in mortality by 3.1 times if native valve endocarditis is treated medically alone without surgical intervention if *S. lugdunensis* is the causative agent [6].

This case presents an additional presentation of pacemaker associated endocarditis with *S. lugdunensis* as the causative agent. One article cites 100 cases of endocarditis secondary to *S. lugdunensis* [7] with another 7 cases of pacemaker associated infective endocarditis from *S. lugdunensis *between 1990 and 2003, four of which were found through the literature review and three within their institutions [1]. *S. lugdunensis* is proving to be a considerable pathogen once introduced into the body with increased virulence compared to other coagulase-negative staphylococci. It will be increasingly important to isolate out different species of the coagulase-negative staphylococci found to be causing systemic infections to further track and identify *S. lugdunensis* and its clinical characteristics.

## 4. Conclusion

For invasive cases of infectious endocarditis, especially those with recently placed hardware, *S. lugdunensis* should be considered on the differential for potential pathogens.
